# A Novel Long-Acting Human Growth Hormone Fusion Protein (VRS-317): Enhanced *In Vivo* Potency and Half-Life

**DOI:** 10.1002/jps.23229

**Published:** 2012-06-07

**Authors:** Jeffrey L Cleland, Nathan C Geething, Jerome A Moore, Brian C Rogers, Benjamin J Spink, Chai-Wei Wang, Susan E Alters, Willem P C Stemmer, Volker Schellenberger

**Affiliations:** 1 Versartis, Inc.Redwood City, California; 2 Amunix, Inc.Mountain View, California; 3 Pacific BioDevelopmentSan Bruno, California

**Keywords:** growth hormone, long acting, growth hormone deficiency, pharmacodynamics, pharmacokinetics, safety, hormones, protein delivery, glomerular filtration

## Abstract

A novel recombinant human growth hormone (rhGH) fusion protein (VRS-317) was designed to minimize receptor-mediated clearance through a reduction in receptor binding without mutations to rhGH by genetically fusing with XTEN amino acid sequences to the N-terminus and the C-terminus of the native hGH sequence. Although *in vitro* potency of VRS-317 was reduced approximately 12-fold compared with rhGH, *in vivo* potency was increased because of the greatly prolonged exposure to the target tissues and organs. VRS-317 was threefold more potent than daily rhGH in hypophysectomized rats and fivefold more potent than daily rhGH in juvenile monkeys. In juvenile monkeys, a monthly dose of 1.4 mg/kg VRS-317 (equivalent to 0.26 mg/kg rhGH) caused a sustained pharmacodynamic response for 1 month equivalent to 0.05 mg/kg/day rhGH (1.4 mg/kg rhGH total over 28 days). In monkeys, VRS-317, having a terminal elimination half-life of approximately 110 h, was rapidly and near-completely absorbed, and was well tolerated with no observed adverse effects after every alternate week subcutaneous dosing for 14 weeks. VRS-317 also did not cause lipoatrophy in pig and monkey studies. VRS-317 is currently being studied in GH-deficient patients to confirm the observations in these animal studies. © 2012 Wiley Periodicals, Inc. and the American Pharmacists Association J Pharm Sci 101:2744–2754, 2012

## INTRODUCTION

Recombinant human growth hormone (rhGH) has been used in the treatment of growth hormone deficiency (GHD) in adults and children since 1986. GHD patients lack the natural endogenous pulsatile secretion of hGH, and therefore, rhGH is a replacement therapy. Current rhGH therapy requires daily subcutaneous (s.c.) injections of rhGH resulting in poor compliance with therapy.^1^ The pharmacokinetic (PK) profile of daily rhGH injections does not mimic the endogeneous multiple daily pulses of hGH, but has been demonstrated for over 30 years to be a safe and effective therapy for treatment of GHD. Clinical studies of continuous infusion of rhGH with a pump demonstrate comparable growth velocity and insulin-like growth factor I (IGF-I) levels to those achieved with daily rhGH injections.^2^–^4^ A continuous infusion of rhGH has been compared with daily rhGH therapy in adult GHD patients for 6 months.^5^ This study indicated that the safety profile and effects on the IGF-I responses were not significantly different between patients treated with continuous infusion of rhGH and daily rhGH therapy. Therefore, continuous, as well as daily, administration of rhGH appears to be safe and efficacious.

Several long-acting rhGH products have been studied in GHD patients over the past 15 years. The first approved long-acting rhGH product, Nutropin Depot® (Genentech, Inc, South San Francisco, California, USA), is no longer marketed because of the difficulties with manufacturing of the product. Nutropin Depot® provides a sustained release of rhGH from a biodegradable polymer similar to that used in resorbable sutures.^6^ The levels of serum hGH sustained in pediatric GHD patients after a single dose of Nutropin Depot® are not sufficient to stimulate growth for the projected monthly dosing interval.^7^ Another long-acting depot formulation of rhGH consists of rhGH encapsulated in hyaluronic acid polymer (LB03002). LB03002 is designed for weekly dosing and provides a sustained rhGH level sufficient to stimulate a pharmacodynamics (IGF-I) response for approximately 5 days after a single dose.^8^ LB03002 has recently been demonstrated to have comparable efficacy to daily rhGH when administered at higher total rhGH in both GHD children^9^ and adults.^10^

PEGylation (the attachment of polyethylene glycol, PEG) has also been used to extend the half-life of rhGH enabling weekly dosing. The first PEGylated rhGH studied in GHD patients (PHA-794428) caused local injection-site lipoatrophy in GHD children resulting in safety concerns^11^ and discontinuation of its development. A similar PEGylated rhGH (NNC126-0083) does not provide sustained pharmacodynamics responses for 1 week after administration in GHD children^12^ and is no longer being developed.

A limitation of hGH therapies and long-acting forms of rhGH such as PEGylated rhGH products is the two clearance mechanisms for hGH, glomerular filtration and receptor-mediated clearance in the kidneys.^13^–^16^ PEGylated or other modified forms of hGH are not subject to glomerular filtration. However, these modified forms are still able to bind the hGH receptor in the kidney and undergo endocytosis. After endocytosis in the kidney, the hGH or modified hGH may be completely or partially metabolized by proteases resulting in excretion of the breakdown products.

VRS-317 was designed to reduce the clearance of rhGH from the body by both glomerular filtration and receptor-mediated clearance using XTEN technology. XTEN technology uses long unstructured hydrophilic sequences of amino acids to increase the half-life of peptides and proteins.^17^ A long N-terminal XTEN sequence is added to rhGH as a fusion protein, increasing the hydrodynamic size of the rhGH, and thereby, reducing glomerular filtration. A C-terminal XTEN sequence is also added to potentially reduce receptor-mediated clearance by decreasing receptor binding. VRS-317 was designed with the identical rhGH sequence present in approved rhGH products, and therefore, contains the identical high- and low-affinity receptor binding epitopes present in approved rhGH products.

## MATERIAL AND METHODS

### Cloning of VRS-317

First, a long XTEN polypeptide (XTEN1) was introduced into the pET30 plasmid (EMD Millipore, Madison, Wisconsin). The hGH gene, corresponding to the native hGH sequence, was amplified by polymerase chain reaction and cloned into the above plasmid at the C-terminus of the XTEN gene. To generate the final VRS-317 expression construct, a short XTEN polypeptide (XTEN2; 13.3 kDa) was appended to the C-terminus of the hGH gene. The final construct, XTEN1–hGH–XTEN2, was transformed into BL21-Gold(DE3), where expression of the fusion protein, VRS-317, could be induced with the addition of isopropyl-β-d-1-thiogalactopyranoside. The XTEN1–rhGH (105.6 kDa) construct was cloned with the same methodology without the addition of the XTEN2.

### Expression and Purification of VRS-317 and XTEN1-rhGH

VRS-317 was expressed in the cytoplasm of *Escherichia coli* as a soluble fusion protein-containing hGH with XTEN1 at the N-terminus and a XTEN2 at the C-terminus. The sequence of rhGH in VRS-317 was identical to human-pituitary-derived hGH (somatotropin). The soluble product was initially recovered by lysis of the host cells. The majority of the host cell proteins and microbial DNA were selectively precipitated by adjusting the pH of the lysate to 4.5 with acetic acid. The acidified lysate was then clarified by a combination of centrifugation and filtration. VRS-317 was then purified by weak anion-exchange chromatography at pH 4.5. Following weak anion-exchange chromatography, the pH of the pooled elution fractions was adjusted to 4.2, the conductivity of the feed was reduced by dilution with water, and then further purified by cation-exchange chromatography. The cation-exchange elution fractions were diluted and the pH was adjusted to greater than 7.5. Product-related aggregates were resolved from the product with additional anion-exchange purification. Finally, the product was formulated and concentrated using tangential flow filtration.

XTEN1–rhGH was also produced usng a similar process as described above.

#### Biophysical Characterization of VRS-317

Nonreducing sodium dodecyl sulfate polyacrylamide gel electrophoresis (SDS-PAGE) analysis of VRS-317 was performed on NuPAGE Bis–Tris 4%–12% polyacrylamide gels (Life Technologies, Carlsbad, California) using NuPAGE MES SDS Running Buffer (Life Technologies). Size exclusion high-performance liquid chromatography (HPLC) analysis of VRS-317 was performed on a BioSEP-SEC-S 4000 7.8 × 600 mm^2^ column (Phenomenex, Torrance, California) using a Shimadzu HPLC system (Shimadzu, Columbia, Maryland).

#### In Vitro Receptor Binding

To evaluate the binding affinity of the XTEN1–rhGH fusion proteins, including VRS-317, a receptor binding enzyme-linked immunosorption assay (ELISA) was performed. The wells of a Costar 3690 plate were coated with 2 μg/mL of recombinant human growth hormone receptor Fc chimera (hGHR-Fc; R&D Systems, Minneapolis, Minnesota), and then thoroughly blocked with a solution of 3% bovine serum albumin (BSA) in phosphate-buffered saline (PBS). Purified XTEN1–rhGH or VRS-317 was serially diluted in Binding Buffer (PBS containing 0.05% Tween-20 and 1% BSA), and incubated on the hGHR-Fc coated wells for at least 1 h at room temperature. Unbound XTEN1–rhGH or VRS-317 was removed by washing with PBS containing 0.05% Tween-20. A biotinylated polyclonal antihGH antibody was used to detect the bound XTEN1–rhGH or VRS-317. Streptavidin–horseradish peroxidase (HRP) was then added, excess removed by washing, and bound amount measured by colorimetric change after addition of a 3,3′,5,5′-tetramethylbenzidine (TMB) peroxidase substrate.

#### Potency Assay

The procedure uses a murine pro-B cell line (BaF/803) that has been stably transfected using hGHR cDNA and pNeo plasmid.^18^ This cell line designated BaF/hGHR/B2B2, expresses approximately 3000 surface hGH receptors per cell, and is hGH responsive (as measured by the stimulation of cellular division). Growth and maintenance of this cell line require the inclusion of either rhGH or interleukin 3 into the media during passaging. The mitogenic response to rhGH is quantified by alamar Blue® to detect the proliferation of the BaF/hGHR/B2B2 cells. The effective concentration to cause 50% of maximum growth (EC50) over 2 days is determined from a minimum of eight different concentrations of the test sample or the positive control, rhGH, in triplicate.

#### PK Experiments

All animal experiments were performed with the approval of MPI Research's Institutional Animal Care and Use Committee and in accordance with accepted standards of humane animal care. Male Sprague–Dawley rats (six per group) were dosed s.c. with 0.3 mg/kg (13.5 nmol/kg) rhGH, 1.5 mg/kg (13.2 nmol/kg) XTEN1–rhGH, or 1.5 mg/kg (12.6 nmol/kg) VRS-317. XTEN1–rhGH and VRS-317 were dosed intravenously (i.v.) to measure the terminal half-life of each protein.

Male cynomolgus monkeys (four per group) were used to assess the PK of the XTEN1–rhGH and VRS-317 after a single s.c. dose of 5 mg/kg XTEN1–rhGH (44.1 nmol/kg) or VRS-317 (42.1 nmol/kg). The plasma samples were taken at predose and at selected time points up to 14 days postdose.

The rate of absorption and bioavailability (absolute) of VRS-317 after s.c. or intramuscular (i.m.) injection was determined in male cynomolgus monkeys (three to four per group) after dosing with 1.5 mg/kg (12.6 nmol/kg) VRS-317 by i.v., i.m., or s.c. administration. Plasma samples were taken predose and at selected time points postdose up to 21 days (504 h).

To assess dose linearity, cynomolgus monkeys were assigned to groups of six to eight males and six to eight females, each receiving s.c. injections of placebo or 1, 5, or 25 mg/kg VRS-317 every 2 weeks for a total of eight injections as part of a good laboratory practice (GLP) toxicology study. Blood samples were collected from animals before dosing and at 0.25, 1, 2, 6, 12, 24, 48, 96, 168, 240, and 312 h after the first dose.

Plasma VRS-317 concentrations were determined by Intertek Alta Analytical Laboratory (San Diego, California) using a validated ELISA with a range of 1–500 ng/mL. Plasma concentrations versus time curves were generated for each animal. All PK data were then plotted as a mean and standard deviation for each group of animals receiving the same dose of VRS-317 (or rhGH). Noncompartmental analysis was performed with WinNonlin professional version 5.2.1 (Pharsight Corporation, St. Louis, Missouri). Concentrations below the quantitation limit of the assay were set to zero for the estimation of parameters. The area under the curve from time zero to the last measurable time point (AUC_0–*t*_) was estimated using the linear trapezoid method. Linear regression over the last three or more time points was used to estimate the elimination rate constant, which was used to estimate terminal half-life (*t*_1/2_) and AUC from zero to infinity (AUC_0–*∞*_). The maximum concentration (*C*_max_) and the time it was observed (*T*_max_) were determined directly from the data.

#### ELISAs for PK

A quantitative sandwich ELISA technique was developed to measure VRS-317 or XTEN1–rhGH in rat and cynomolgus monkey plasma. In the assay, standards, controls, and test samples are incubated with anti-XTEN immunoglobulin G antibody that has been immobilized on a microtiter plate. After incubation, unbound material is washed away and VRS-317 or XTEN1–rhGH is detected using biotinylated goat anti-human GH antibody followed by streptavidin–HRP and visualized with a TMB peroxidase substrate. The assay has a range of 1–500 ng/mL.

rhGH PK was determined from plasma samples using an ELISA Kit purchased from R&D Systems.

#### Hypophysectomized Rat Studies

Sprague–Dawley male rats weighing 70–100 g (six per group) had their pituitaries surgically removed. The placebo, rhGH, and VRS-317 were administered via a s.c. injection in the cervical region on the dorsal surface. Body weights were measured and recorded daily for several days before dosing. Rats were randomized into treatment groups to insure balance for average body weight across the groups.

Necropsy examinations were performed under procedures approved by a veterinary pathologist on all animals to determine the level of pituitary ablation due to the surgery. Both tibias were collected from all animals and fixed in 10% neutral buffered formalin, decalcified, embedded in paraffin, sectioned with a microtome into approximately 4 μm sections, stained with toluidine blue and examined with a light microscope. Each tibia was cut at five progressive levels at approximately 150–200 μm intervals, thus, yielding five epiphyseal sections for measurements. Data were examined by analysis of variance with a Tukey–Kramer posthoc test for pairwise comparisons using JMP software (SAS Institute, Cary, North Carolina) and a *p* value of 0.05.

#### Juvenile Cynomolgus Monkey Study

Young adult (approximately 2 years of age) cynomolgus monkeys were assigned to one of four treatment groups (two males/two females per group). VRS-317 was administered 0.4 or 1.4 mg/kg on Days 1, 29, and 57. Additional groups received placebo or rhGH (Saizen®; Merck-Serono, Geneva, Switzerland) at a dose of 0.05 mg/kg daily from Days 1 to 28. A dose volume of 0.28 mL/kg was employed in each of the VRS-317 dose groups, whereas the rhGH group was dosed at a volume of 0.05 mL/kg. During the study, cageside observations were recorded daily, body weights were measured weekly, and the injection site was evaluated and scored for erythema and edema at times, which corresponded with blood collections for PK at several time points (predose and up to 672 h postdose). Blood was collected from VRS-317 animals for clinical pathology evaluations (hematology and/or clinical chemistry) pretreatment and 24 h after dosing on Days 1, 29, and 57 and on Day 85. Two 5 mm punch biopsies of injection sites were obtained from animals treated with VRS-317, 5 days following dosing on Days 29 and 57. Fat content from the injection sites was evaluated and graded. The PK of rhGH and VRS-317 was measured after the first and last dose. IGF-I and IGF binding protein 3 (IGFBP-3) analyses were performed at several time points between each dose and after the last dose. Body weight and food consumption was monitored daily. Immune responses (antibodies) to VRS-317, hGH, and XTEN were measured prestudy, during study, and at completion of the study (Day 85).

#### Pharmacodynamic Assays

Insulin-like growth factor I analysis for adult cynomolgus monkeys was performed on plasma samples after acid ethanol extraction using an immunoassay in a Luminex® bead format (Milliplex IGF-1 kit; EMD Millipore Corporation, Billerica, Massachusetts). IGF-I and IGFBP-3 analyses for juvenile cynomolgus monkeys were performed using an immunoassay (Siemens Immulite 2000; Pacific Biomarkers, Inc., Seattle, Washington).

The dose proportionality of the pharmacodynamics response to VRS-317 was studied after s.c. administration in male cynomolgus monkeys. Monkeys (*n* = 4/group) were dosed with 0.3, 1.5, or 7.5 mg/kg VRS-317 (2.5, 12.6, and 63 nmol/kg, respectively). Plasma samples for IGF-I levels were taken predose and at selected time points up to 21 days (504 h) postdose. The response to treatment (VRS-317 or rhGH) was calculated using each animals baseline IGF-I or IGFBP-3 and the percent increase from baseline in each animal. All pharmacodynamic data were then plotted as a mean and standard deviation for each group of animals receiving the same dose of VRS-317 or rhGH.

#### Safety Assessment in Cynomolgus Monkeys

A total of 28 male (3.15–4.2 kg) and 28 female (2.2–2.7 kg) naïve cynomolgus monkeys were assigned to groups to receive s.c. injections of placebo or VRS-317 every 2 weeks for a total of eight injections. Four groups of three animals per sex per group received the placebo or VRS-317. The dose levels of 0, 1.0, 5.0, and 25.0 mg/kg VRS-317 were given at respective dose volumes of 0.758, 0.030, 0.152, and 0.758 mL/kg. Two additional animals of each sex were included in the placebo and 25.0 mg/kg dose group for a 28-day recovery period following the dosing period to assess the persistence, reversibility, and/or delayed occurrence of any potential treatment effects. The dosing formulations were injected between the skin and underlying layers of tissue to one of two injection sites in the scapular region on the back of each animal, rotating the injection sites at each dose.

Observations for morbidity, mortality, injury, and the availability of food and water were conducted twice daily. Detailed clinical observations (including an evaluation of injection sites) were conducted weekly. Body weights were measured and recorded weekly beginning Day −1. Ophthalmoscopic examinations were conducted pretest and before each necropsy.

Physical examinations were conducted pretest. Electrocardiographic examinations and respiration rate determinations were conducted pretest and at approximately 1–2 h postdose on Day 99. Blood and urine samples for clinical pathology evaluations were collected pretest and on Days 100 and 128. At necropsy on Days 100 and 128, macroscopic examinations were performed, organ weights were recorded, and a comprehensive list of tissues was collected for microscopic examination from all animals terminated on Day 100 and the injection site on Day 128.

#### Pig Lipoatrophy Study

Four healthy naïve female Sinclair miniature swine were acclimated for at least 3 days before assignment to this study. Each animal assigned to study received s.c. injections of a placebo, 1.0 mg rhGH, and 5.4 and 27 mg VRS-317 to separate injection areas designated on the dorsal area of the animal. Following the initial injections, injections of 1.0 mg rhGH were administered to all animals at approximately 8 h intervals for the duration of the study. PK blood samples and skin biopsies were collected up to 120 h from the initial injection. Animals were sacrificed following their final designated blood and biopsy collection time point. Biopsy samples were measured by quantitative morphometry and histomorphometry.

## RESULTS

### Expression and Purification

**Figure 1:**
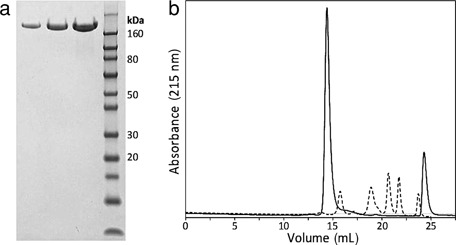
(a) SDS-PAGE of 2, 5, and 10 μg of VRS-317 compared with a molecular weight marker. (b) Size exclusion HPLC analysis of VRS-317 (solid curve) compared with a set of globular molecular weight standards (dashed curve, Bio-Rad Laboratories, Hercules, California). The standard includes thyroglobulin 670 kDa, gamma-globulin 158 kDa, ovalbumin 44 kDa, myoglobin 17 kDa, and vitamin B12 1.35 kDa. The formulation buffer for VRS-317 elutes at 25 mL.

VRS-317 (XTEN1–rhGH–XTEN2) was expressed as a fusion protein of rhGH and hydrophilic, unstructured amino acids, XTEN. The XTEN domains of VRS-317 increased the solubility and stability of the rhGH domain. Unlike rhGH, VRS-317 was soluble in the cytoplasm of *E. coli* and no inclusion bodies were observed. Purification was facilitated by the increased acid stability of VRS-317. After cell lysis, treatment with acetic acid caused precipitation of most host cell proteins with minimal VRS-317 loss. The negative charge from the glutamate residues in the XTEN domain resulted in a p*I* of approximately 3 for VRS-317, as compared with the p*I* of 5.2 for rhGH.^19^ These properties enabled the use of ion-exchange methods to remove host cell proteins, DNA, and aggregates of VRS-317. As shown in Figure 1, the final purified material was a single band on a nonreducing SDS-PAGE gel slightly larger than its calculated molecular weight (MW) of 119 kDa and did not contain aggregates as measured by size exclusion chromatography. The large size and unstructured nature of the XTEN domains provide a large hydrodynamic radius as noted by the early elution time in the size exclusion chromatogram,^17^ as confirmed by size exclusion chromatography with multiangle light scattering (112,290 Da; data not shown). The large hydrodynamic radius introduced by the XTEN sequence and the low p*I* of VRS-317 also cause VRS-317 to migrate less than the globular protein markers on the SDS-PAGE gel (e.g., gamma-globulin, 158 kDa, p*I* ∼ 6.9). The XTEN1–rhGH construct was expressed and purified yielding a similar high-purity fusion protein for further characterization.

### Molecule Optimization

**Figure 2:**
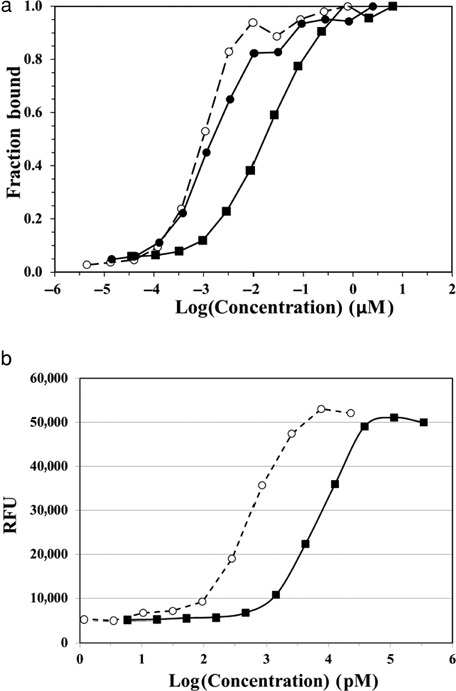
(a) Receptor-binding ELISA results for rhGH(•; dashed line), XTEN1–rhGH (▪), and VRS-317 (▪). (b) *In vitro* cell-based potency assay results for rhGH (•; dashed line) and VRS-317 (▪) [relative fluorescence units (RFU) measuring number of viable cells].

An early construct of VRS-317 was designed to maximize the binding to the hGH receptor *in vitro*. This construct was a fusion protein of a long XTEN sequence at the N-terminus (83.6 kDa) and rhGH at the C-terminus (XTEN1–rhGH; 105.6 kDa). To assess the impact of reduced receptor binding, a short XTEN domain (XTEN2; 13.3 kDa) was added to the C-terminus of rhGH along with the long XTEN (XTEN1) domain at the N-terminus. There was no significant effect of the long N-terminal XTEN (XTEN1–rhGH) on binding affinity when compared with rhGH in an ELISA-based receptor binding assay. However, VRS-317 (XTEN1–rhGH–XTEN2) had an approximately 11-fold reduction in affinity (IC50 = 27 nM) compared with rhGH (IC50 = 2.5 nM) (Fig. 2a). The ability of VRS-317 to bind to two hGH receptors and cause dimerization that results in intracellular signaling was tested using a cell line engineered to proliferate in response to rhGH. In this *in vitro* potency assay, the EC50 of VRS-317 was 12-fold (6.8 nM) greater than rhGH (0.6 nM) indicating a lower *in vitro* potency for VRS-317 (Fig. 2b).

Because the XTEN constructs had significantly larger molecular mass than rhGH, the analyses below were performed using both mass (mg/kg) and molar (nmol/kg) dosing to provide an estimate of the rhGH dose administered in each case (rhGH MW: 22 kDa). VRS-317 (119 kDa) had a molecular mass approximately fivefold greater than rhGH.

### Pharmacokinetics

**Figure 3:**
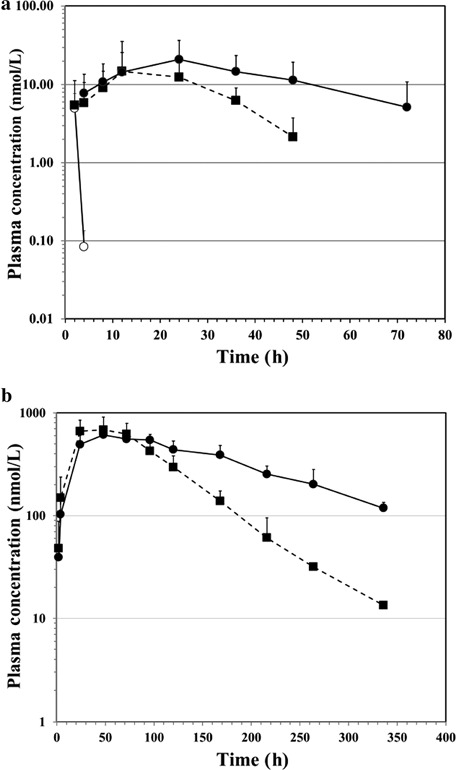
(a) Pharmacokinetics in rats after subcutaneous (s.c.) administration of 0.3 mg/kg (13.5 nmol/kg) rhGH (•), 1.5 mg/kg (13.2 nmol/kg) of XTEN1–rhGH (▪; dashed line), or 1.5 mg/kg (12.6 nmol/kg) of VRS-317 (▪). (b) The pharmacokinetics of XTEN1–rhGH (▪; dashed line; 44.1 nmol/kg) and VRS-317 (▪, 42.1 nmol/kg) after a single 5 mg/kg s.c. injection in adult cynomolgus monkeys.

To assess the PK of VRS-317 and XTEN1–rhGH, studies were performed in rats and monkeys (Fig. 3). Rats were dosed s.c. with approximately equal molar doses of rhGH, XTEN1—rhGH, or VRS-317. In addition, XTEN1–rhGH and VRS-317 were dosed i.v. to measure the *t*_1/2_ of each protein. As shown in Figure 3a, rhGH has a rapid clearance after s.c. administration. XTEN1–rhGH and VRS-317 had a slower absorption phase, greater *C*_max_, and a longer *t*_1/2_ than rhGH. The terminal elimination half-lives of XTEN1–rhGH and VRS-317 determined after i.v administration in rats were 6.8 and 15 h, respectively. The PK of XTEN1–rhGH and VRS-317 was also determined in monkeys after a single s.c. dose. As shown in Figure 3b, both the XTEN1–rhGH and VRS-317 achieved a similar *C*_max_ within 48 h postdose, but VRS-317 had a significantly longer half-life (XTEN1–rhGH: 48.6 h; VRS-317: 110 h).

**Figure 4:**
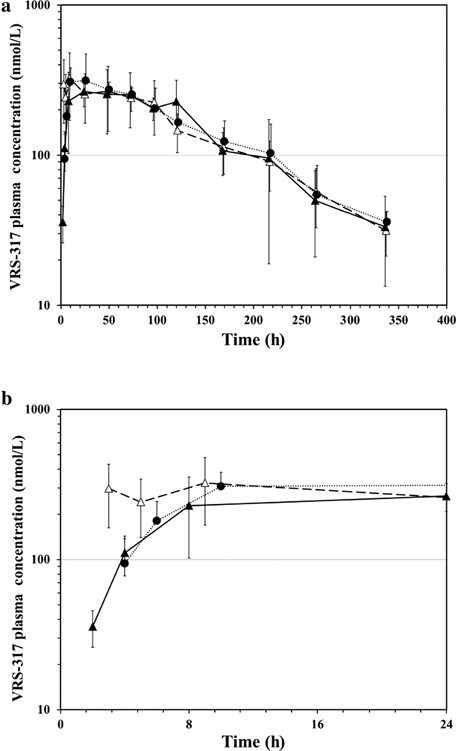
(a) Pharmacokinetics of 1.5 mg/kg (12.6 nmol/kg) of VRS-317 in cynomolgus monkeys dosed either intravenously (i.v.; ▵), subcutaneously (s.c.; ▴) or intramuscularly (i.m.; ▪). (b) Same results shown for first 24 h after dosing.

To determine the rate and extent of absorption of VRS-317 after s.c. or i.m. injection, the PK of VRS-317 was studied in monkeys after i.v. (*n* = 4), s.c. (*n* = 3) or i.m. (*n* = 3) administration of 1.5 mg/kg (12.6 nmol/kg) VRS-317. VRS-317 was rapidly absorbed after either s.c. or i.m. administration (Fig. 4). The *C*_max_ relative to that following i.v. injection was 78.5% and 89% for s.c. and i.m., respectively. The bioavailability based on AUC_0–*t*_ was 101% and 103% for s.c. and i.m., respectively. These results demonstrated that VRS-317 was rapidly and near completely absorbed after s.c. administration.

The dose proportionality of the PKs of VRS-317 was studied after s.c. administration in cynomolgus monkeys as part of a safety study. The PK profile of VRS-317 after single s.c. administration to monkeys was approximately linear with dose (Table 1). These results indicate that the absorption and clearance of VRS-317 was not dependent upon dose level between 1 and 25 mg/kg (8.4–210 nmol/kg).

**Table 1:** Pharmacokinetic Parameters (Mean ± SD) Estimated by Noncompartment Methods After Single Subcutaneous Doses of VRS-317 in Cynomolgus Monkeys

Dose (mg/kg) (nmol/kg)	*C*_max_ (μg/mL)	*C*_max_ (nM)	AUC_0–*∞*_ (μg h/mL)	AUC_0–*∞*_ (nM h)
1 (8.4)	24.8 ± 13.5	208 ± 114	2046 ± 488	17,190 ± 4099
5 (42)	98.8 ± 65.2	830 ± 548	11,004 ± 3861	92,471 ± 32,441
25 (210)	476 ± 236	3,999 ± 1985	87,911 ± 26,155	738,751 ± 219,787

**Figure 5:**
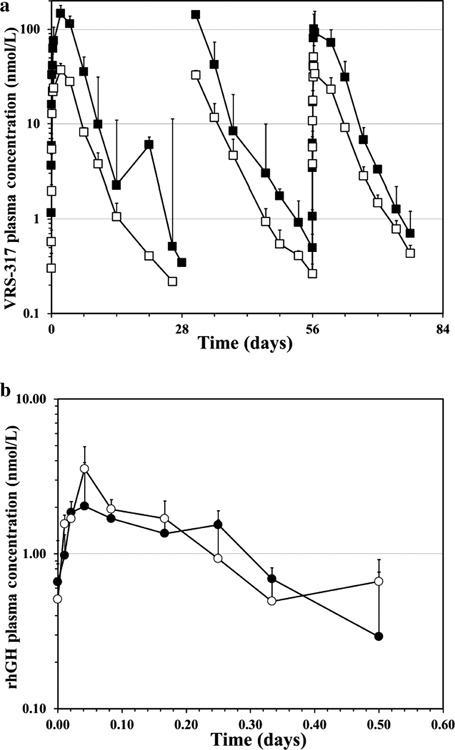
(a) Pharmacokinetics of VRS-317 dosed s.c. at 0.4 (□) or 1.4 (▪) mg/kg (3.4 or 11.8 nmol/kg) VRS-317 every 28 days in juvenile cynomolgus monkeys (genders combined). (b) Pharmacokinetics of 0.05 mg/(kg day) rhGH dosed s.c. in juvenile cynomolgus monkeys after first (Day 1; ▪) and last dose (Day 28; •).

Repeat dose PK was evaluated in juvenile cynomolgus monkeys (two per sex per group) administered s.c. doses of 0.4 or 1.4 mg/kg (3.4 or 11.8 nmol/kg) VRS-317 every 28 days or 0.05 mg/kg/day rhGH for 28 days. The PKs of VRS-317 were determined after each dose and the PKs of rhGH were determined after first (Day 1) and last (Day 28) dose as shown in Figure 5. A consistent and dose proportional *C*_max_ (0.4 mg/kg: 40 ± 17 nM; 1.4 mg/kg: 100 ± 34.5 nM) was noted after each administration. No accumulation was noted on the basis of comparable *C*_max_ and exposure (AUC-0.4 mg/kg: 4440 ± 1209 nM-h; 1.4 mg/kg: 16800 ± 3586 nM-h) after each administration.

### Pharmacodynamics

Weight gain in a hypophysectomized-rat-administered daily rhGH for 6 or 7 days was the standard historical method for assessment of rhGH potency. To assess the *in vivo* potency of VRS-317, different dose regimens of VRS-317 were compared with a fixed dose of daily rhGH in hypophysectomized rats dosed s.c.

In the first experiment, VRS-317 dosed every other day at 0.34 mg/kg (12 nmol/kg total) produced the same total weight gain as daily dosing of 0.10 mg/kg hGH (35 nmol/kg total). There was also no difference in the thickness of the tibial epiphyses between the two treatment groups (Table 2). Higher doses of VRS-317 produced an increased weight gain and tibial epiphyses thickness compared with daily rhGH treatment. These results indicated a greater *in vivo* potency of VRS-317 relative to rhGH on a molar basis. In addition, VRS-317 demonstrated a direct effect on bone growth in this model.

**Table 2:** Potency Assessment of Different Doses and Regimens of VRS-317 Compared with rhGH and Placebo in Hypophysectomized Rats

Dose (mg/kg) [hGH equiv mg/kg]	Dose (nmol/kg)	Dosing Frequency	Total Dose (nmol/kg)	*C*_ss_*a* (nM)	Body Weight Gain (g; ±SD)–Day 7	Tibial Epiphyses Width (μm; ±SD)
*Experiment 1*						
Placebo	0	Days 1, 3, 5, and 7	NA*b*	NA	0.8 ± 1.1	617 ± 158
rhGH control: 0.1	5	Daily	35	NA	10.7 ± 0.8*	905 ± 51*
VRS-317: 5.40 [1.0]	45	Days 1 and 5	90	57.1	13.7 ± 1.1*	915 ± 60*
VRS-317: 1.35 [0.25]	11	Days 1, 3, 5, and 7	44	10.0	14.3 ± 1.1*	917 ± 39*
VRS-317: 0.34 [0.06]	3	Days 1, 3, 5, and 7	12	1.34	9.7 ± 2.3*	854 ± 80*
*Experiment 2*						
Placebo	0	Daily (1–6)	NA	NA	2.0 ± 2.4	574 ± 152
rhGH control: 0.1	5	Daily (1–6)	30	NA	12.3 ± 4.5	884 ± 262*
VRS-317: 0.34 [0.06]	3	Days 1, 3, and 5	9	1.34	15.2 ± 7.3*	1046 ± 316*
VRS-317: 0.057 [0.01]	0.5	Daily (1–6)	3	0.54	4.8 ± 1.2	731 ± 197
VRS-317: 0.17 [0.03]	1.5	Daily (1–6)	9	1.63	15.3 ± 5.6*	1101 ± 308*

a *C*_ss_, steady-state VRS-317 concentration calculated from mean of simulated PK for Day 1–7 (Day 1, first dose day).

b NA, Not Applicable.

* Significantly different from placebo, *p* < 0.05.

To evaluate the relationship between VRS-317 plasma levels and potency in hypophysectomized rats, an additional dose ranging study was performed comparing lower doses of VRS-317 with daily-administered rhGH. A total dose of 1 mg/kg (9 nmol/kg) of VRS-317 [3 nmol/kg every other day or 1.5 nmol/kg per day] produced a weight gain slightly greater than a total hGH dose of 35 nmol/kg (Experiment 2, Table 2). Similar effects were also noted on the thickness of tibial epiphyses after sacrifice of the rats on Day 7. Using single dose PK data following s.c. administration of VRS-317 or rhGH in rats, the relationship between plasma exposure and efficacy in hypophysectomized rats was modeled to calculate the steady-state plasma concentration (*C*_ss_) of VRS-317. These results indicate that sustaining a level of VRS-317 above 1 nM provides a growth response comparable to a daily dose of rhGH in hypophysectomized rats.

**Figure 6:**
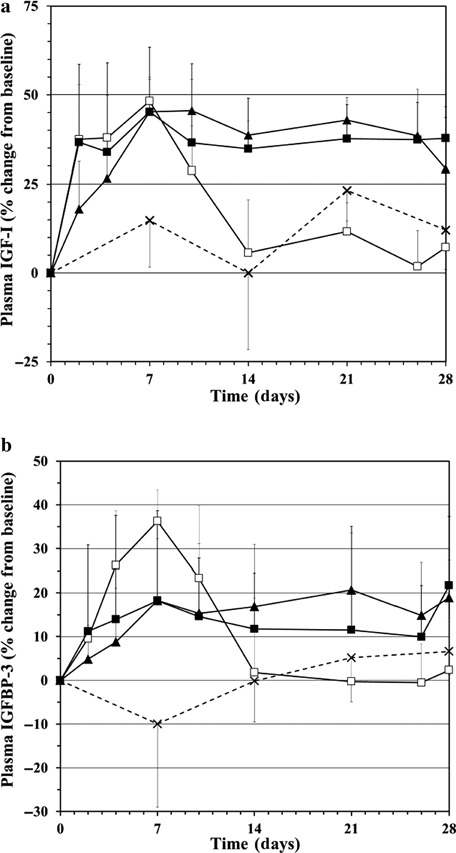
IGF-I (a) and IGFBP-3 (b) changes from baseline in juvenile cynomolgus monkeys after administration of placebo (×), 0.05 mg/(kg day) rhGH for 28 days (▴; 1.4 mg/kg rhGH; 64 nmol/kg), 0.40 (□) or 1.4 mg/kg (▪; 3.4 or 11.8 nmol/kg, respectively) VRS-317 as single s.c. injection.

The effect of sustained VRS-317 exposure compared with daily rhGH administration was also assessed in juvenile cynomolgus monkeys. As shown in Figure 6, 1.4 mg/kg (11.8 nmol/kg) VRS-317 stimulated an IGF-I and IGFBP-3 response comparable to 0.05 mg/kg/day rhGH in juvenile cynomolgus monkeys over 28 days (Total rhGH dose: 1.4 mg/kg or 64 nmol/kg). The pharmacodynamic response was achieved at approximately a sixfold lower molar dose than daily hGH indicating that the sustained levels of VRS-317 stimulated the pharmacodynamic response continuously over the 28 days. The reduced pharmacodynamic response noted around 14 days after dosing of 0.4 mg/kg (3.4 nmol/kg) VRS-317 correlated with the plasma levels of VRS-317 decreasing below 1 nM (119 ng/mL VRS-317; Fig. 5a).

A similar IGF-I response was noted in adult cynomolgus monkeys (four males per group) administered a single s.c. dose of VRS-317. A dose dependent increase in IGF-I levels was noted in monkeys administered 0.3, 1.5, or 7.5 mg/kg (2.5, 12.6, or 63 nmol/kg, respectively) VRS-317 with mean maximum increases above baseline (predose) of 71%, 204%, and 335%.

### Safety Assessment of VRS-317

To date, VRS-317 has been administered to rats, pigs, and monkeys as single doses and to rats and monkeys as repeat doses up to 25 mg/kg (63 nmol/kg). No observable clinical signs or symptoms have been noted in these studies.

In the juvenile cynomolgus monkey study, no test article-related effects among lipid parameters were identified in any group at any interval. All individual animal variations were considered to be within the range of normal biologic variability for this species. Therefore, no adverse effects were noted in monkeys dosed with 0.40 or 1.4 mg/kg VRS-317 every 28 days for 84 days. At Day 85, two monkeys dosed 1.4 mg/kg VRS-317 developed low titer antibodies against VRS-317 that were specific to the rhGH domain. This antibody response was probably caused by the lack of homology between cynomolgus GH and rhGH. No anti-XTEN antibodies were detected. There were no detectable differences in VRS-317 PK or the pharmacodynamics response in these monkeys, suggesting that the low titer antibody responses did not have a significant clinical effect.

In the 14-week GLP monkey toxicology study, monkeys were administered placebo or 1, 5, or 25 mg/kg VRS-317 every other week. There were no VRS-317-related effects on the following parameters: clinical observations, dermal irritation scores, body weights, ophthalmoscopic examinations, respiratory rate, electrocardiographic parameters, coagulation parameters, clinical chemistry analytes, urinalysis parameters, macroscopic observations, or organ weights. There were no adverse histopathology changes attributed to VRS-317. In particular, no significant differences in glucose or lipid metabolism, mammary or prostate tissues, or brain tissues were noted between placebo and VRS-317 treated animals. None of the effects noted during this study were considered adverse. Therefore, the No-Observed-Adverse-Effect Level (NOAEL) for this study was 25.0 mg/kg per dose of VRS-317. The proposed highest dose in the ongoing single administration Phase 1 trial in adult GHD patients is 0.8 mg/kg (based on a 70 kg human), which on a human equivalent dose basis is approximately 10-fold below the NOAEL established in this repeat-dose toxicology study in monkeys.

Because lipoatrophy has been noted for PEGylated rhGH, the potential for lipoatrophy caused by s.c. administration of VRS-317 was extensively studied in pigs and monkeys. Four healthy naïve female Sinclair miniature swine were injected s.c. at three injection-site grids per pig (12 total grids). In each injection-site grid, there was a placebo control, an rhGH control (injected every 8 h), 20 mg methylprednisolone, or 5.4 or 27 mg of VRS-317. Injection sites were harvested by biopsy from 24 to 96 h postdose. Histology and histomorphometry were conducted on each biopsy sample. There were no visible changes except for the rhGH control in which inflammation and tissue damage resulted from repeated injections. There was no significant lipoatrophy observed for any of the treatments at any of the time points by morphometry. These data suggest that VRS-317 does not cause overt lipoatrophy.

No lipoatrophy has been noted following the administration of VRS-317 in the monkey studies conducted to date. Injection-site biopsies from the repeat dose juvenile monkey study were analyzed to further assess the potential for VRS-317 to cause lipoatrophy. The fat content of skin biopsies from the injection sites was quantitatively evaluated. Differences in fat content appeared related to individual and/or sampling variations, and there were no discernible VRS-317 and/or time effect. Overall the qualitative evaluation correlated to the quantitative histomorphometric evaluation. In addition, injection-site analyses from the 14-week GLP monkey toxicology study also did not reveal any notable lipoatrophy.

## DISCUSSION

Previous attempts to develop long-acting rhGH products focused on either continuous delivery of native rhGH or modifications such as PEGylation without altering the binding of rhGH to its receptor. These approaches only achieved short-term (weekly or less) rhGH exposure and subsequently lower pharmacodynamic (e.g., IGF-I) and/or growth responses.

By contrast, VRS-317 was designed to have a lower affinity for the hGH receptor, thereby reducing receptor-mediated clearance and, in turn, potentially minimizing side effects such as lipoatrophy. By attaching the hydrophilic amino acid sequences, XTEN, to the N-terminus (XTEN1) and C-terminus (XTEN2) of rhGH, the receptor binding and cell-based potency of VRS-317 was reduced approximately 11–12-fold compared with rhGH (Fig. 2). By contrast, the XTEN1–rhGH construct with only XTEN on the N-terminus of rhGH had comparable receptor binding to rhGH (Fig. 2a). The increased half-life of VRS-317 compared with XTEN1–rhGH (Fig. 3) cannot be explained by the small difference in molecular mass (VRS-317: 119 kDa; XTEN1–rhGH: 105.6 kDa) and may be the result of the reduction in receptor binding for VRS-317 compared with XTEN1–rhGH. These studies demonstrated that reducing rhGH binding affinity increased terminal elimination half-life.

A concern with reduced *in vitro* potency was the potential need for higher doses of VRS-317 compared with rhGH. However, the molar potency *in vivo* of VRS-317 was at least threefold greater than daily rhGH as noted by the bone growth and weight gain in hypophysectomized rats (Table 2). A fivefold lower molar dose of VRS-317 was required to provide sustained pharmacodynamic responses (IGF-1 and IGFBP-3) compared with daily rhGH in juvenile rhesus monkeys (Fig. 6). These studies indicated that a lower rhGH dose in the form of VRS-317 was required to invoke pharmacodynamic and growth responses presumably because of the longer tissue exposure time to rhGH when administered as VRS-317.

The PK of VRS-317 was dose proportional (Table 1) and reproducible (Fig. 4), allowing for evaluation of the relationship between the plasma concentration of VRS-317 and the biological responses. Sustaining a VRS-317 at 1 nM or above was sufficient to achieve a sustained growth response in hypophysectomized rats (Table 2) and pharmacodynamic responses in monkeys (Fig. 6). The magnitude of the pharmacodynamic responses in monkeys was dose proportional ensuring that a dose ranging study in humans may allow for accurate prediction of an efficacious dose.

Historical studies of other rhGH formulations as well as continuous infusion studies have indicated that transient glucose and lipid metabolism changes may occur.^4^,^5^ In a 14-week GLP toxicology study in adult monkeys and a 12-week study in juvenile monkeys, there were no observed changes in these clinical parameters. Only low-titer hGH-specific immune responses that did not affect the PK or pharmacodynamics of VRS-317 were noted after repeated dosing (juvenile monkeys 1.4 mg/kg VRS-317 every 28 days). In addition, VRS-317 was well tolerated at the maximum dose administered (25 mg/kg) every other week in the GLP toxicology study with no observable adverse effects noted by detailed and comprehensive clinical observations or detailed histological examinations.

Other long-acting forms of rhGH have caused injection-site lipoatrophy.^11^ The lipoatrophy may have occurred because of a sustained s.c. exposure to rhGH, which stimulates lipolysis. VRS-317 has reduced affinity for the hGH receptor, and therefore, may bind less to the local hGH receptors in the s.c. tissues. In addition, VRS-317 was rapidly absorbed after s.c. administration reducing the potential for lipoatrophy. A lipoatrophy study was conducted in pigs and no lipoatrophy was noted with VRS-317. In addition, no lipoatrophy has been noted to date in the monkey studies, some of which included injection-site histology.

Overall, VRS-317 is a novel rhGH construct with the potential to be administered up to monthly in GHD adults and children at total monthly doses lower than the typical daily rhGH. VRS-317 may lead to increased compliance and convenience in GHD patients resulting in improvements in therapeutic benefits without increased safety concerns.
